# Genetic architecture of left ventricular noncompaction in adults

**DOI:** 10.1038/s41439-020-00120-y

**Published:** 2020-10-15

**Authors:** Samantha Barratt Ross, Emma S. Singer, Elizabeth Driscoll, Natalie Nowak, Laura Yeates, Rajesh Puranik, Raymond W. Sy, Sulekha Rajagopalan, Alexandra Barratt, Jodie Ingles, Richard D. Bagnall, Christopher Semsarian

**Affiliations:** 1 Agnes Ginges Centre for Molecular Cardiology, Centenary Institute, The University of Sydney, Newtown, Australia; 2 Sydney Medical School, Faculty of Medicine and Health, The University of Sydney, Newtown, Australia; 3 Wiser Healthcare, Sydney, Australia; 4 Department of Cardiology, Royal Prince Alfred Hospital, Sydney, Australia; 5 Department of Clinical Genetics, Liverpool Hospital, Liverpool, Australia; 6 Sydney School of Public Health, Faculty of Medicine and Health, The University of Sydney, Sydney, Australia

**Keywords:** Genetics research, Genetic testing

## Abstract

The genetic etiology and heritability of left ventricular noncompaction (LVNC) in adults is unclear. This study sought to assess the value of genetic testing in adults with LVNC. Adults diagnosed with LVNC while undergoing screening in the context of a family history of cardiomyopathy were excluded. Clinical data for 35 unrelated patients diagnosed with LVNC at ≥18 years of age were retrospectively analyzed. Left ventricular (LV) dysfunction, electrocardiogram (ECG) abnormalities, cardiac malformations or syndromic features were identified in 25 patients; 10 patients had isolated LVNC in the absence of cardiac dysfunction or syndromic features. Exome sequencing was performed, and analysis using commercial panels targeted 193 nuclear and mitochondrial genes. Nucleotide variants in coding regions or in intron-exon boundaries with predicted impacts on splicing were assessed. Fifty-four rare variants were identified in 35 nuclear genes. Across all 35 LVNC patients, the clinically meaningful genetic diagnostic yield was 9% (3/35), with heterozygous likely pathogenic or pathogenic variants identified in the *NKX2-5* and *TBX5* genes encoding cardiac transcription factors. No pathogenic variants were identified in patients with isolated LVNC in the absence of cardiac dysfunction or syndromic features. In conclusion, the diagnostic yield of genetic testing in adult index patients with LVNC is low. Genetic testing is most beneficial in LVNC associated with other cardiac and syndromic features, in which it can facilitate correct diagnoses, and least useful in adults with only isolated LVNC without a family history. Cardiac transcription factors are important in the development of LVNC and should be included in genetic testing panels.

## Cardiomyopathy: Genetic testing largely unhelpful for rare heart disease

Genetic testing is of limited use for diagnosing a rare heart muscle disorder called left ventricular non-compaction (LVNC), a structural abnormality of the lower left chamber of the heart in which the muscular wall appears soft and spongy rather than smooth and compacted. A team led by Christopher Semsarian from the University of Sydney, Australia, carried out a a retrospective analysis of genetic data from 35 patients diagnosed with LVNC but with no family history of the disease. They identified only three likely pathogenic gene variants, all in known heart regulatory genes and all in individuals with symptoms of cardiac dysfunction. The researchers conclude that genetic testing can facilitate a correct diagnosis of LVNC in patients showing signs of heart problems, but is largely unhelpful for those without symptoms and no family history.

## Introduction

Left ventricular noncompaction (LVNC) is an arrhythmogenic cardiomyopathy characterized by prominent left ventricular (LV) trabeculations with deep intertrabecular recesses and thinning of the compact epicardium. LVNC is increasingly being diagnosed in adults, though the clinical significance of LVNC presenting in adulthood remains unclear, particularly when the diagnosis is made outside the context of an affected family^1^. LVNC is classified as an inherited cardiomyopathy of autosomal dominant inheritance, and clinical screening of first-degree family members is indicated^2^. Outside such families, LV trabeculation identified in an individual may be an incidental finding or a feature of a syndrome^3,4^. Major adverse events include life-threatening arrhythmias, thromboembolism, and cardiac failure.

Identification of a genetic etiology is important because it can aid in correct diagnoses, identify at-risk family members and inform reproductive decisions. Studies of the genetic causes of LVNC have primarily identified variants in cardiomyopathy genes, including *MYH7*, *MYBPC3* and *TTN*, with reported genetic testing yields between 17 and 41%^5^. The diagnostic genetic yield in these studies may be biased by the inclusion of individuals screened in the context of a family history of cardiomyopathy, for which the genetics are better understood. Genetic testing is typically performed using clinical LVNC gene panels that screen a large number of cardiomyopathy-associated genes. Exome sequencing has the added advantage of facilitating analysis of nuclear and mitochondrial genomes as well as reassessment of data when new gene-disease associations are elucidated. Both nuclear and mitochondrial genome variants have been associated with LVNC^6^.

In this study, we sought to assess the value of genetic testing in adult index patients diagnosed with LVNC outside the context of family screening and to explore the potential of genetic testing to guide the clinical diagnosis and management of such individuals.

## Materials and methods

### Study cohort

This study included all unrelated index patients diagnosed with LVNC in adulthood, defined as >18 years of age, who attended the Genetic Heart Disease Clinic, a cardiac tertiary referral centre at the Royal Prince Alfred Hospital in Sydney, Australia, between 2002 and 2018. Blood used for genetic testing was available for all enroled subjects. The patients were previously diagnosed with LVNC in the context of symptomatic or asymptomatic assessment and were referred for evaluation and management. Patients who attended the clinic for cardiac screening in the context of a significant family history were excluded. Informed consent was obtained, and the Sydney Local Health District Ethics Committee approved the study.

### Clinical assessment

**Fig. 1:**
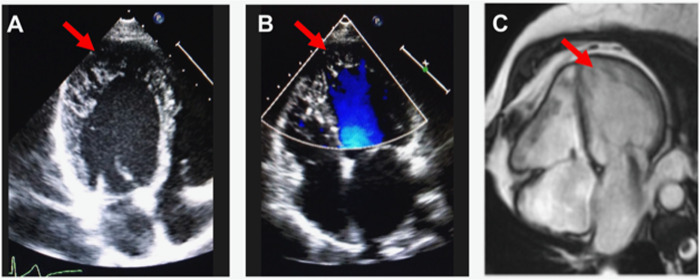
Echocardiogram and cardiac magnetic resonance imaging in LVNC. Left ventricular non-compaction from a representative subject from the study, on **a**, 2D-transthoracic echocardiogram, **b**, 2D-transthoracic echocardiogram with colour Doppler flow, and **c**, cardiac magnetic resonance imaging. Red arrows indicate regions of non-compaction.

Confirmatory diagnoses were made by experienced cardiologists based on clinical presentation and interpretation of echocardiogram and cardiac magnetic resonance imaging results (Fig. 1) using the Jenni and Petersen criteria, respectively^7,8^. Past clinical data were retrospectively retrieved from medical records: clinical assessment included physical examination, 12-lead electrocardiogram (ECG), and echocardiogram. LV systolic dysfunction was defined as a systolic left ventricular ejection fraction (LVEF) < 50% on echocardiogram. Cardiac magnetic resonance imaging results were available for the majority of the patients. The patients were also assessed for any syndromic features, including pectus excavatum and upper limb abnormalities.

### Genetic investigation

Exome sequencing of DNA isolated from whole blood with QIAamp DNA Mini Kit (Qiagen, Hilden, Germany) was performed using the Illumina NovaSeq platform. The genetic investigation was primarily limited to 193 genes included in clinically available genetic testing panels offered by Invitae, Blueprint genetics, Ambry, and GeneDx (Supplementary Table 1). The 193 genes comprise 180 nuclear-encoded genes and 13 mitochondrially encoded genes. Nuclear-encoded genes were considered in the context of autosomal dominant or X-linked inheritance with an allele frequency of <0.004% or autosomal recessive inheritance with an allele frequency <1% in gnomAD, as previously described^9,10^. Ultrarare variants were defined as those with a gnomAD allele count = 0.

The mitochondrial genome was sequenced using off-target exome sequencing reads, and variants were annotated with MitoMaster^11,12^. The analysis was initially restricted to variants in mitochondrial genes included in the clinical panels, as follows: *MTND1, MTND5, MTTD, MTTG, MTTH, MTTI, MTTK, MTTL, MTTL2, MTTM, MTTQ, MTTS1*, and *MTTS2*. In subsequent analysis, rare mitochondrial variants of all 37 mitochondrially encoded genes were assessed.

Nuclear gene variants were assessed according to the American College of Medical Genetics and Genomics (ACMG) guidelines^13^. Variants that failed to reach likely pathogenic or pathogenic classifications were considered rare and had supportive in silico tool predictions; genotype-phenotype associations were considered to be highly suspicious variants of uncertain significance (VUS). Mitochondrial variants were compared with the Cambridge reference sequence (CRS). A variant was considered potentially disease causing if it was not haplotype associated and had an overall allele frequency of <0.05% according to frequency data in GenBank, which contains 49,135 full-length human mitochondrial sequences^14^. MitoTip scores based on frequency, the type of nucleotide change, and nucleotide conservation were taken into consideration as variants in tRNA-encoding genes^15^. All rare nuclear and mitochondrial variants are reported (Supplementary Table 2).

## Results

### Characteristics of LVNC patients

A total of 35 LVNC index patients were included (Table 1); of these, 25 had LVNC and LV systolic dysfunction, ECG abnormalities or cardiovascular malformations, and 10 had LVNC in the absence of LV systolic dysfunction, ECG abnormalities or cardiovascular malformations. The 35 unrelated LVNC patients were predominantly Caucasian (*n* = 33, 94%) and female (*n* = 19, 54%), with a mean age at diagnosis of 43.8 ± 12.3 years (range 21–77 years). Seventy-four percent of the patients (*n* = 26) were diagnosed following a symptomatic presentation; nine patients were diagnosed incidentally. Patients had LV dilation (*n* = 10, 29%), asymmetric LV hypertrophy (*n* = 1, 3%) and atrial septal defects (*n* = 5, 14%). Extracardiac manifestations included skeletal muscle weakness (*n* = 1, 3%), pectus excavatum (*n* = 2, 6%) and upper limb abnormalities (*n* = 2, 6%). Half of the patients were taking aspirin (*n* = 18, 51%), and 26% (*n* = 9) had an in situ cardioverter defibrillator implanted.

**Table 1:** Clinical characteristics of LVNC cohort.

	All patients (*n* = 35) *n*, (%)
Female	19 (54)
Caucasian (European) ethnicity	33 (94)
Mean age at diagnosis (yrs)	43.8 ± 12.3
NYHA class I	29 (83)
NYHA class II	6 (17)
LV systolic dysfunction (LVEF < 50%)	12 (34)
LVEF (%)	53.1 ± 13.4
Hypertension	7 (20)
Presentation	
Symptomatic (including palpitations, exertional dyspnoea, syncope and atrial fibrillation)	26 (74)
Incidental	9 (26)
Familial disease	14 (40)
Left ventricular non-compaction	4 (11)
Valvular disease	2 (6)
Septal defect	2 (6)
Hypertrophy	2 (6)
Dilated cardiomyopathy	3 (9)
Polycystic kidney disease	2 (6)
Holt-Oram syndrome	2 (6)
Muscle weakness	1 (3)
Sudden cardiac death	1 (3)
Structural features	
Left atrial dilation	19 (54)
Left ventricle dilation	10 (29)
Prominent RV trabeculation	13 (37)
LVEDV (Indexed)	90.63 ± 20.88
LVESV (Indexed)	40.33 ± 21.21
RVEDV (Indexed)	89.77 ± 16.10
RVESV (Indexed)	39.18 ± 12.64
Resting and ambulatory ECG findings	
Non-sustained ventricular tachycardia	7 (20)
Atrial fibrillation	12 (34)
Left bundle branch block	6 (17)
Right bundle branch block	5 (14)
Surgical/medical interventions	
Aspirin	18 (51)
Pacemaker	2 (6)
Implantable cardioverter defibrillator	9 (26)

*LVEDV* left ventricular end-diastolic volume, *LVESV* left ventricular end-systolic volume, *RVEDV* right ventricular end-diastolic volume, *RVESV* right ventricular end-systolic volume, *EF* ejection fraction, *LV* left ventricle, *LVEF* left ventricular ejection fraction.

### Genetic findings in LVNC patients

Fifty-four rare variants in 35 nuclear genes were identified (Supplementary Table 2). Ultrarare nuclear variants were identified in 18 patients (51%). Three patients (9%) had pathogenic or likely pathogenic variants in *NKX2*-*5* and *TBX5* (Table 2). Interestingly, no pathogenic or likely pathogenic variants were identified in clinically unaffected individuals with no family history.

**Table 2:** Likely pathogenic, pathogenic and highly suspicious variants of uncertain significance identified.

Gene	gnomAD count	Variant consequence	DNA/protein alteration	Missense constraint score (z)	pLI	ClinVar	ACMG prediction	Patient
**Likely pathogenic/pathogenic variants in nuclear genes**								
*NKX2-5*	0	SNV (stop gained)	NM_004387.3:c.744 C > A/ NP_004378.1:p.Tyr248Ter	1.84	0.86	NR	Pathogenic (PVS1, PM2, PP3)	BHS
	0	INDEL (frameshift)	NM_004387.3:c.677_680del/ NP_004378.1:p.Asp226AlafsTer5			NR	Likely pathogenic (PVS1, PM2)	CBT
*TBX5*	0	INDEL (Frameshift)	NM_000192.3:c.105dup/ NP_000183.2:p.Ser36GlnfsTer25	1.63	0.99	NR	Pathogenic (PVS1, PM2, PP3)	BRP
**Highly suspicious variants of uncertain significance in nuclear genes**								
*ACTC1*	0	Missense (possible splice)	NM_005159.4:c.723 C > G/ NP_005150.1:p.Ser241Arg	5.25	0.95	NR	VUS (PM2, PP2, PP3)	BLM
*DSP*	0	INDEL	NM_001008844.1:c.2598_2603del/ NP_001008844.1:p.Trp867_Gln868del	0.91	1	NR	VUS (PM2)	SS
*HCN4*	0	SNV (missense)	NM_005477.2:c.1438 G > A/ NP_005468.1:p.Gly480Ser	4.83	0.23	NR	VUS (PM2, PP3)	ME
*MYBPC 3*	0	SNV (missense)	NM_000256.3:c.989 C > T/ NP_000247.2:p.Pro330Leu	0.69	0	NR	VUS (PM1, PM2, PP3)	BLU
*MYH6*	4	SNV (missense)	NM_002471.3:c.756 C > G/ NP_002462.2:p.His252Gln	2.87	0	537949	VUS (PP3)	BSQ
*PRDM16*	0	INDEL (frameshift)	NM_022114.3:c.564del/ NP_071397.3:p.Ser189ValfsTer22	2.06	1	NR	VUS (PM2, PP3)	ALW
*TBX5*	0	Intron (splice region)	NM_000192.3:c.510 + 5 G > T/-	1.63	0.99	NR	VUS (PM2, PP3)	BEW
*TPM1*	0	SNV (missense)	NM_000366.5:c.647 A > G/ NP_000357.3:p.Gln216Arg	3.42	0.8	NR	VUS (PM2, PP2, PP3)	AXT
**Highly suspicious variants of uncertain significance in mitochondrial genes**								
*MT-TV*	N/A	SNV	c.1612C > T	–	–	–	–	BKS

*SNV* single nucleotide variant, *INDEL* insertion-deletion, *NR* not reported, *VUS* variant of uncertain significance. Positive missense constraint scores (z) indicate that less variants are observed in the particular gene than expected. *pLi* probability of loss of function intolerance. pLi scores close to 1 indicate that a gene cannot tolerate protein-truncating variation. *ACMG* American College of Medical Genetics.

**Fig. 2:**
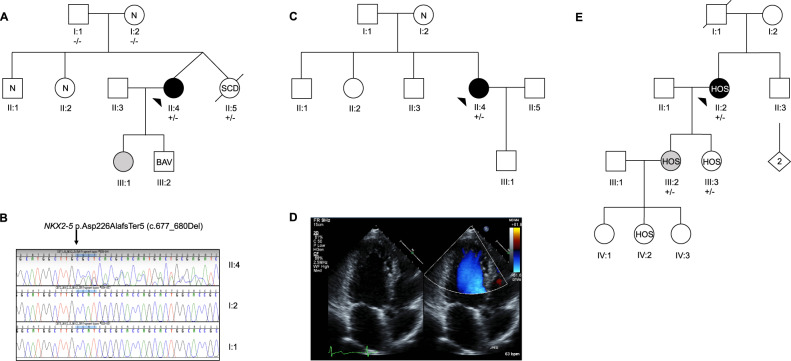
Pedigrees for three families; two with loss of function variants in *NKX2-5*, and one with a loss of function variant in *TBX5*. **a** CBT family; II:5 experienced a sudden cardiac death (SCD), III:1 has a small segment of posterior LVNC non-compaction, III:2 has a bicuspid aortic valve (BAV), **b** Sanger sequencing revealing de novo *NKX2-5* variant in II:4, **c** BHS family, **d** 2-D transthoracic echocardiogram with colour Doppler flow from BHS proband II:4, and **e** BRP family; II:2 has LVNC and Holt-Oram syndrome (HOS), III:2 has HOS and increased left ventricular trabeculation/possible LVNC, III:3 has HOS, IV:2 has HOS. Grey symbol = increased left ventricular trabeculation/possible LVNC; +/− = heterozygous for variant; −/− = normal; arrow indicates proband.

Female index patient CBT1 (Fig. 2a) was diagnosed with LVNC following fatigue and increasing chest pain at 36 years of age. Clinical assessment revealed LV systolic dysfunction, diffuse hypokinesis, a repaired atrial septal defect, and NSVT. The de novo frameshift variant p. Asp226AlafsTer5 was identified in *NKX2-5* (Fig. 2b). Two VUS including the nonsense variant p. Arg4991Ter in *TTN* and the missense variant p. Leu709Phe in *DSP* were identified. The family review revealed a son with a bicuspid aortic valve and a deceased twin sister who experienced sudden death at 19 years of age but with no structural abnormalities identified on autopsy. Genetic analysis of a postmortem sample from the deceased twin sister identified the p. Asp226AlafsTer5 variant in *NKX2-5*.

Female index patient BHS1 (Fig. 2c, d) was incidentally diagnosed with LVNC at 34 years of age following screening for a volunteering program and was subsequently found to have marked first-degree AV block and later developed VT. Genetic analysis revealed a nonsense variant, p. Tyr248Ter, in exon 2 of *NKX2-5*. A review of her family revealed no overtly affected relatives.

Female index patient BRP1 was diagnosed with LVNC at 49 years of age, and her symptoms included general fatigue, chest tightness, palpitations and presyncope. Clinical examination revealed brachydactyly, ankylosing spondylitis and pectus deformity. ECG and Holter monitor investigations revealed sinus node dysfunction, intermittent sinus pauses and evidence of isorhythmic dissociation. Assessment of family members revealed three generations of triphalangeal thumbs (Fig. 2e). Genetic analysis revealed the frameshift variant, p. Ser36ThrfsTer25 in *TBX5*^16^, and she was subsequently diagnosed with Holt-Oram syndrome (HOS). Cosegregation analysis identified two daughters who were heterozygous for the *TBX5* variant, and both were diagnosed with HOS. ECG parameters and echocardiography were within normal limits in one daughter aged 25 years at the time of this study. Echocardiography revealed mild LV dilation, apical hypokinesis and possible LVNC in the other daughter aged 30 years. One granddaughter was found to have upper limb skeletal abnormalities consistent with a clinical diagnosis of HOS.

### Mitochondrial genome analysis

**Fig. 3:**
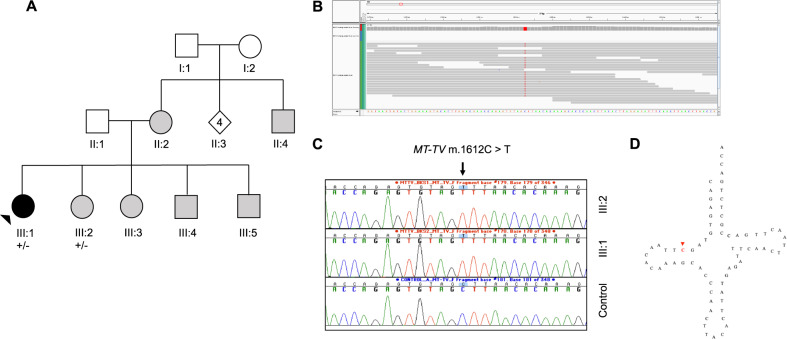
Family with mitochondrial variant in *MT-TV* gene. **a** BKS family pedigree, **b** variant identified in whole-exome sequencing data, **c** Sanger sequencing revealing variant in tRNA-valine, **d** variant position in mitochondrial tRNA valine. III:1 has neurological and cardiac involvement (LVNC and hypertrophy), II:4 experienced stroke at 21 years of age, III:4 and III:5 have muscle weakness and had developmental delay, III:2 has epilepsy and has experienced unexplained syncopal events. Grey symbols = individuals who have shown features consistent with mitochondrial disease however have not received a diagnosis.

Ten mitochondrial haplotypes were identified, with haplotype H (*n* = 15, 43%) being the most common (Supplementary Table 2). Overall, 16 patients (46%) had a VUS in the mitochondrial genome. One likely causal variant, c.1612C > T, in *MT-TV* was identified in the mitochondrial genome (Fig. 3). The variant position shows 100% interspecies conservation and is predicted to be likely pathogenic by MitoTIP^15^. Female index patient BKS was diagnosed with LVNC, with coexisting septal hypertrophy and an atrial septal defect, at 21 years of age after presenting with atypical chest pain. Her clinical history was significant for developmental delay, life-long muscle weakness, sensorineural hearing loss and autonomic dysfunction. A sister who also carries the variant had a tilt table test consistent with postural tachycardia syndrome complicated by neurocardiogenic syncope, without evidence of cardiomyopathy.

## Discussion

In recent years, the number of adults diagnosed with LVNC has increased, though the benefit of genetic testing is unclear among patients^1^. We show in a small LVNC cohort that the diagnostic genetic yield was low (9%) among adult index patients who present outside the context of family screening. All causative variants were identified in those patients with overt disease, suggesting that genetic testing is likely to be most beneficial in LVNC associated with cardiac dysfunction or malformations and noncardiac systemic features and least useful in clinically unaffected adults with only isolated LVNC.

To our knowledge, this is the first study to assess a large number of LVNC genes (193 genes) using a targeted exome sequencing approach in adult index patients. In contrast to previous studies, multiple variants in *MYBPC3* and *MYH7* cardiomyopathy genes were not identified^5^. This difference likely reflects the exclusion of patients presenting in the context of a family history of cardiomyopathy. Interestingly, all three pathogenic or likely pathogenic variants were identified in genes encoding transcription factors. In addition, we identified highly suspicious VUS in a heterogeneous group of genes, suggesting that many different mechanistic pathways may result in a noncompaction phenotype and highlighting the importance of using a broad genetic testing panel.

Our findings add to existing evidence that suggests an important association between transcription factor variants, specifically in *NKX2*-5 and *TBX5*, and LVNC. A small number of human genetic studies have identified causative variants in *NKX2-5*, an important regulator of cardiac trabeculation and conduction system development in LVNC^17,18^. Analysis of 50 patients carrying *NKX2-5* variants showed that AV block is the most consistent phenotype, and patients are at risk of sudden death even in the absence of significant cardiomyopathy^18^. A recent study has shown that mice with conditional knockout of *NKX2-5* develop excessive trabeculation associated with conduction defects and heart failure progression^19^. Assessment of induced pluripotent stem cell-cardiomyocytes from LVNC patients with the stop-gain variant p. Tyr317Ter in *TBX20*, encoding a transcription factor, highlights its regulation of *PRDM16* and how the loss of *PRDM16* regulation can result in LVNC^20^. Thus, we provide further evidence for the causative role of the *PRDM16* loss-of-function variant identified in the present cohort and the important role of the cardiac transcription factor network in the pathogenesis of LVNC.

Twenty-six percent of clinically affected patients in this study were heterozygous for highly suspicious VUS, which highlights the lack of genotype-phenotype evidence guiding pathogenicity classifications in LVNC^3^. This issue is exemplified by the classification of the previously mentioned loss-of-function variant in *PRDM16* as a VUS. Loss-of-function mutations in *PRDM16* have previously been found in patients with LVNC, DCM and sudden cardiac death; however, more evidence is required for these variants to be considered a known disease mechanism, which would facilitate a likely pathogenic or pathogenic classification^5^. Furthermore, there is increasing evidence supporting the role of *ACTC1* variants in the pathogenesis of LVNC, and it is likely that the *ACTC1* p. The Ser241Arg variant is the cause of disease in index patient BLM^21^.

The requirement of a large gene panel relates to the lack of standardized diagnostic criteria, which results in a heterogeneous patient cohort for which variant interpretation is difficult. Indeed, thorough clinical phenotyping can aid variant interpretation, and in many cases, it is the coexisting feature that guides classification. The identification of a *DSP* deletion is more meaningful in the context of LVNC with dyskinesis and scarring. The classification of loss-of-function variants in *TBX5* relies on the identification of upper limb abnormalities consistent with Holt-Oram syndrome (HOS)^16^. Although there is minimal evidence, namely, in the form of case reports, identifying LVNC in the context of *TBX5* variants is mechanistically plausible given the high prevalence of other structural abnormalities, including septal and valvular defects^22^. Additional studies are required to better understand whether the association of LVNC with *TBX5* variants and HOS represents a true mechanistic link or an incidental finding. As the use of genetic testing in LVNC increases, we are seeing the development of stronger gene-disease associations, including that of LVNC and variants in *HCN4*^5,23^. We identified three patients with rare missense variants in *HCN4* despite the conserved nature of the gene. Of particular interest is variant p. Gly480Ser, which may be located in a hotspot as p. Gly480Arg; it has previously been described in a large family with sinus bradycardia^24^. However, it is difficult to determine whether variants identified in adult patients are related to increased trabeculation or coexisting cardiac abnormalities; it is possible that hypertrabeculation distracts from the true phenotype. If we can improve phenotype accuracy, it may be feasible to reduce the size of the panel screened, which would have the added benefit of reducing the number of VUSs that are identified.

A review of the mitochondrial genome, facilitated by our exome sequencing approach, revealed a likely monogenic *MT-TV* gene cause of disease in a patient with a mixed LVNC and hypertrophy phenotype. Variants in *MT-TV* have previously been associated with HCM^25^. Across the cohort, a small number of rare mitochondrial variants were identified and might be associated with the LVNC phenotype. A previous study has shown that mitochondrial variants may operate in tandem with nuclear variants in the pathophysiological process that underlies LVNC^6^.

**Fig. 4:**
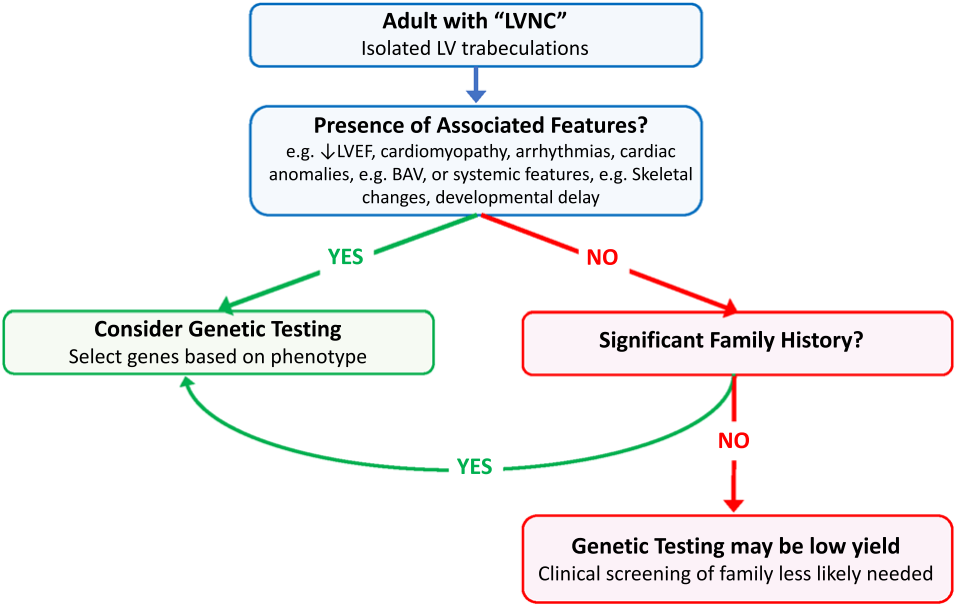
Summary of key study findings illustrating the clinical settings where genetic testing may be useful in patients with LVNC. LVNC Left Ventricular Noncompaction, LV left ventricular, LVEF left ventricular ejection fraction.

Our findings have implications for clinical practice. The current body of work highlights that performing genetic testing for all LVNC index patients is likely to result in a low diagnostic yield and that the greatest utility is for individuals with LV dysfunction, ECG abnormalities or syndromic/coexisting clinical features (Fig. 4). For these patients, identification of a pathogenic variant can provide confidence in the diagnosis and heritability of the condition, and individuals can benefit from prenatal diagnostic options, carrier testing and genetic counselling. We demonstrate how the use of a comprehensive gene panel can facilitate clarification of previously unclear diagnoses, such as Holt-Oram syndrome. Furthermore, the inclusion of transcription factors in gene panels is important for the identification of genetic etiologies of LVNC. However, outside a research setting, comprehensive gene panels should be used with caution given the tendency for identification of a large number of VUSs. Genetic testing is likely to be least useful, with a potential for no benefit, in adults with isolated LVNC with no cardiac dysfunction, syndromic abnormalities or family history.

The main limitation in studying the genetic etiology of LVNC stems from issues with the diagnostic criteria that result in highly heterogeneous patient cohorts. As such, our small cohort of 35 index cases may not be representative of the general population. Although we identified a potentially causal variant in an additional 26% of patients, only three (9%) carried variants with sufficient evidence to classify as likely pathogenic or pathogenic. By utilizing exome sequencing, it is possible that we missed variants in deep intronic regions, as well as large rearrangements and karyotype abnormalities. Moreover, genetic testing results were not available for all first-degree members in this study, and therefore lack of cosegregation is a limitation of this study. This limitation is particularly highlighted by the lack of variant cosegregation in the BHS and BKS families.

Genetic testing applied broadly to adult index patients with LVNC is likely to have a low diagnostic yield. This work suggests that genetic testing is likely to be most beneficial in LVNC associated with other cardiac features, such as LV dysfunction, other cardi and noncardiac syndromic features, and least useful in adults with only isolated LVNC in the absence of cardiac dysfunction and syndromic features. Although the diagnostic yield of genetic testing was low in this study for adult index patients with LVNC who did not present in the context of family screening, genetic testing can aid in facilitating correct diagnoses and appropriate patient management. The results from this study highlight the important role of cardiac transcription factor variants in the development of LVNC. Further research is required to elucidate the pathogenesis of LVNC, which shows significant clinical and genetic heterogeneity, and to confirm our finding that there is minimal benefit of genetic testing in individuals diagnosed with isolated LVNC.

## Supplementary information

Supplementary Data
