# Supplementary material for: Genetic architecture of left ventricular noncompaction in adults
Source: Hum Genome Var. 2020 Oct 15;7:33. doi: 10.1038/s41439-020-00120-y (PMC7566488; doi:10.1038/s41439-020-00120-y)
Supplement: Supplementary file 1 — Supplementary Data [file 41439_2020_120_MOESM1_ESM.docx]

**SUPPLEMENTARY DATA**

**Supplementary Table 1: Nuclear and mitochondrial gene list**

| *ABCA1* | *CRYAB* | *JUP* | *MTTM* | *SCN3B* |
| --- | --- | --- | --- | --- |
| *ABCC9* | *CSRP3* | *KCND3* | *MTTQ* | *SCN4B* |
| *ABCG5* | *CTNNA3* | *KCNE1* | *MTTS1* | *SCN5A* |
| *ABCG8* | *CYP27A1* | *KCNE2* | *MTTS2* | *SGCD* |
| *ACTA2* | *DES* | *KCNE3* | *MURC* | *SKI* |
| *ACTC1* | *DMD* | *KCNH2* | *MYBPC3* | *SL01B1* |
| *ACTN2* | *DOLK* | *KCNJ2* | *MYH11* | *SLC2A10* |
| *ACVRL1* | *DSC2* | *KCNJ5* | *MYH6* | *SMAD3* |
| *AKAP9* | *DSG2* | *KCNJ8* | *MYH7* | *SMAD4* |
| *ALMS1* | *DSP* | *KCNQ1* | *MYL2* | *SNTA1* |
| *ALPK3* | *DTNA* | *KRAS* | *MYL3* | *SOS1* |
| *ANK2* | *EFEMP2* | *LAMA4* | *MYLK* | *SOS2* |
| *ANKRD1* | *EMD* | *LAMP2* | *MYLK2* | *SPRED1* |
| *APOA1* | *ENG* | *LCAT* | *MYOZ2* | *TAZ* |
| *APOA5* | *EPHB4* | *LDB3* | *MYPN* | *TBX1* |
| *APOB* | *EYA4* | *LDLR* | *NEBL* | ***TBX20*** |
| *APOC2* | *FBN1* | *LDLRAP1* | *NF1* | *TBX5^a^* |
| *APOC3* | *FBN2* | *LIPA* | ***NKX2-5*** | *TCAP* |
| *APOE* | *FBX032* | *LMF1* | *NOTCH1* | *TECRL* |
| *BAG3* | *FHL1* | *LMNA* | *NRAS* | *TGFB1* |
| *BGN* | *FKBP14* | *LOX* | *PCSK9* | *TGFB2* |
| *BRAF* | *FKRP* | *LPL* | *PDLIM3* | *TGFB3* |
| *CACNA1C* | *FKTN* | *LRRC10* | *PKP2* | *TMEM43* |
| *CACNA2D1* | *FLNA* | *LZTR1* | *PLEKHM2* | *TMPO* |
| *CACNB2* | *FLNC* | *MAP2K1* | *PLN* | *TNNC1* |
| *CALM1* | ***FOXE3*** | *MAP2K2* | *PLOD1* | *TNNI3* |
| *CALM2* | *GAA* | *MAT2A* | *PPP1CB* | *TNNT2* |
| *CALM3* | ***GATA4*** | *MED12* | *PRDM16* | *TNXB* |
| *CASQ2* | *GATAD1* | *MFAP5* | *PRDM5* | *TOR1AIP1* |
| *CAV3* | *GDF2* | *MIB1* | *PRKAG2* | *TPM1* |
| *CBL* | *GLA* | *MTND1* | *PTPN11* | *TRDN* |
| *CBS* | *GPD1L* | *MTND5* | *RAF1* | *TRPM4* |
| *CHRM2* | *GPIHBP1* | *MTTD* | *RASA1* | *TTN* |
| *CHST14* | *HCN4* | *MTTG* | *RBM20* | *TTR* |
| *COL1A1* | *HFE* | *MTTH* | *RIT1* | *TXNRD2* |
| *COL1A2* | *HRAS* | *MTTI* | *RYR2* | *VCL* |
| *COL3A1* | *ILK* | *MTTK* | *SCN10A* | *ZNF469* |
| *COL5A1* | *JAG1* | *MTTL* | *SCN1B* |  |
| *COL5A2* | *JPH2* | *MTTL2* | *SCN2B* |  |

*Transcription factors that have critical functions in heart development in bold.*

**Supplementary Table 2: Clinical and genetic characteristics of index patients diagnosed with LVNC in adulthood**

| **Patient** | **Age^a^** | **Presentation** | **Non-compaction** | **Rare nuclear gene variants (gnomAD count)** | **Mitochondrial haplotype (total variants)** | **Rare mitochondrial gene variants** | |
| --- | --- | --- | --- | --- | --- | --- | --- |
| JZ | 77 | Symptomatic | Infero-apical LV | *APOB* p.Tyr183Cys (0)  *SNTA1* p.Ala435Val (2)  *TTN* p.Arg3148Leu (1) | I4a (33) | - | |
| ME | 43 | Symptomatic | Infero-apical LV | *HCN4* p.Gly480Ser (0)  *RBM20* p.Gly179Asp (0) | K1a (42) | *MT-ND5* 13665T>C  *COII* 7729A>G* | |
| SS | 48 | Symptomatic | Distal and apical LV | *DSP* p.Trp867_Gln868del (0)  *LDLR* p.Gly137Ser (3) | J1b (37) | - | |
| ALL | 41 | Symptomatic | Apico-lateral and apico-inferior LV | *CAV3* p.Tyr121Asp (0) | W3a (36) | - |  |
| ALW | 23 | Symptomatic | Posterolateral wall and apical LV | *PRDM16* p.Ser189ValfsTer22 (0)  *AKAP9* p.Ser1892Phe (0)  *SOS2* p.Pro799Leu (4) | M6a (41) | NC5 5895C>T*  *MT-RNR1* 978A>G*  *MT-ND1* 3486C>T | |
| ALY | 29 | Symptomatic | Apical LV | - | J1c (30) | *MT-ATP6* 8574C>T  *MT-RNR2* 2905A>G* | |
| APE | 24 | Incidental | Mid-distal LV | *ABCA1* p.Met1988Leu (2)  *MAT2A* c.293-1G>T (7)  *PCSK9* p.Arg97Cys (4)  *RYR2* p.Lys1225Thr (2)  *TTN* p.Tyr3052His (9) | H72 (13) | *COIII* 9261A>G*  *MT-ND5* 13785C>T | |
| AXC | 29 | Symptomatic | Apical LV | *LOX* p.Gly165Arg (10) | K2a (32) | - | |
| BEW | 52 | Symptomatic | Apical LV | *TBX5* c.510+5G>T (0)  *CBL* p.Ser252Phe (0) | H26a (12) | - | |
| BHS | 34 | Incidental | Inferoapical LV | *NKX2-5* p.Tyr248Ter (0)  *KCNJ8* p.Arg274His (9) | T2a (34) | - | |
| BLM | 36 | Symptomatic | Apical LV | *ACTC1* p.Ser241Arg (0) | H1a (15) | *MT-ND1* 3612C>T | |
| BLU | 47 | Symptomatic | Apical and lateral LV | *MYBPC3* p.Pro330Leu (0)  *TTN* p.Gly31043Ser (0) | J1b (36) | - | |
| BRP | 49 | Incidental | Middle to apical LV | *TBX5* p.Ser36GlnfsTer25 (0)  *CACNB2* p.Val60Leu (3)  *TTN* p.Phe5336Tyr (0) | J1c (35) | *MT-CYB* 15373A>G* | |
| BSK | 48 | Incidental | Apical LV | *HCN4* p.Arg999Trp (4)  *SCN3B* p.Arg78Gln (4) | U4a (28) | *MT-CYB* 15693T>C* | |
| BSQ | 34 | Incidental | Inferoapical LV | *MYH6* p.His252Gln (4)  *FBN2* p.Arg1054Pro (0) | K1a (36) | - | |
| BUH | 44 | Symptomatic | Inferolateral wall and apical LV | *TTN* c.8903-104A>G (5) | V10b (16) | - | |
| BVJ | 42 | Symptomatic | Anterolateral, inferolateral walls apical LV | *RBM20* p.Ile1090SerfsTer14 (1)  *COL5A1* p.Gly1613Ser (6) | X2e (30) | - | |
| BVU | 48 | Symptomatic | Midcavity LV | *MYH6* p.Arg654Trp (5)  *LZTR1* p.Gln319ArgfsTer32 (2)  *TTN* p.Ser17823Arg (0)  *TTN* p.Ser19464Arg (0) | H3 (12) | *MT-CYB* 15667C>T*  *CR:HVS3* 503A>G* | |
| AVZ | 51 | Symptomatic | Apical LV | *PCSK9* p.Arg272Trp (13) | K1a (31) | *MT-CYB* 15498G>A * | |
| AXT | 35 | Symptomatic | Inferior wall and apical LV | *TPM1* p.Gln216Arg (0)  *RYR2* c.11881-180A>G (0) | H1e (10) | - | |
| BJV | 54 | Symptomatic | Diffuse | *-* | H4a (15) | - | |
| BKS | 21 | Symptomatic | Distal anterolateral LV | *COL5A2* p.Thr1057Asn (13)  *RYR2* p.Asn658Ser (10) | H5k (12) | *MT-TV* 1612C>T* | |
| BQZ | 54 | Symptomatic |  | - | H82 (11) | - | |
| CBT | 36 | Symptomatic | Anterolateral and apical LV | *NKX2-5* p.Asp226AlafsTer5 (0)  *TTN* p.Arg4991Ter (2)  DSP p.Leu709Phe (6) | K1c (35) | *MT-ND2* 5099C>T*  *COI* 5979 G>A* | |
| CCG | 54 | Incidental | Apical LV | - | H (11) | - | |
| PX | 49 | Symptomatic | Apical LV | - | H5a (11) | - | |
| AHM | 43 | Incidental | Distolateral and apical LV | *RYR2* p.Val2452Met (7) | H1U (13) | - | |
| AZA | 23 | Symptomatic | Apical LV | *COL1A2* p.Pro123His (0)  *SCN5A* p.Arg1023Gly (0) | H51 (11) | - | |
| BPD | 50 | Symptomatic | Apical LV | *HCN4* p.Arg524Gln (5) | H13a (17) | *MT-RNR2* 2557C>T | |
| BRZ | 52 | Symptomatic | Apical LV | - | J1c (33) | *COII* 9219T>G*  *MT-ND4* 11503C>T*  *MT-ND5* 12545C>T | |
| BGM | 56 | Incidental | Apical LV | *NOTCH1* p.Ala71Thr (2) | H1a (12) | - | |
| BHC | 48 | Symptomatic | Distal posterolateral LV wall | *MIB1* p.Arg318Ter (8)  *KCNH2* p.Thr542Lys (1)  *TTN* p.Gly14712Arg (5) | J1c (31) | *MT-ND4L* 10559A>G* | |
| BOG | 38 | Symptomatic | Apical LV | *TTN* p.Glu20800Gly (13) | W5a (37) | - | |
| BYN | 61 | Symptomatic | Distal LV | *AKAP9* p.Thr1357Ile (2)  *SOS2* p.Met985Thr (0)  *TTN* p.Arg3074Gln (2) | U5b (29) | *MT-CYB* 15631A>G* | |
| BFE | 60 | Incidental | Apical and lateral walls LV | - | HV9 (11) | *MT-ND5* 14145A>G | |

*^a^Patient age at diagnosis; *variant in gene not included on gene panel*
